# First description of *Arthroderma lilyanum* in a rabbit with a focal alopecic area of the forelimb

**DOI:** 10.1016/j.mmcr.2023.04.003

**Published:** 2023-05-11

**Authors:** Julia Lienhard, Nicole Wengi, Ana Rostaher, Marianne Schneeberger, Giovanni Ghielmetti

**Affiliations:** a Section of Veterinary Bacteriology, Institute for Food Safety and Hygiene, Vetsuisse Faculty, University of Zurich, Zurich, 8057, Switzerland; b Clinic for Small Animal Internal Medicine, Vetsuisse Faculty, University of Zurich, Zurich, 8057, Switzerland; c Dermatology Unit, Clinic for Small Animal Internal Medicine, Vetsuisse Faculty, University of Zurich, Zurich, 8057, Switzerland

**Keywords:** *Arthroderma lilyanum*, Dermatophytosis, Rabbit

## Abstract

Dermatophytosis is an important zoonotic disease in pet rabbits. While common clinical signs of dermatophytosis can occur, rabbits can also be asymptomatically infected. This case report describes a rabbit from Switzerland, with a focal alopecic area on one forepaw. Dermatophyte culture of a hair and skin sample taken from the lesion revealed growth of a dermatophyte, that was identified as the recently described species *Arthroderma (A.) lilyanum* by sequencing of the internal transcribed spacer (ITS) and β-tubulin genes. After local treatment with a disinfectant containing octenidine dihydrochloride and phenoxyethanol twice daily for two weeks, the lesion fully healed. Although it is not clear whether the dermatophyte was responsible for the lesion or if it was an incidental finding with an asymptomatic infection, the current report shows, that the host spectrum and geographical distribution of *A. lilyanum* are broader than previously thought.

## 1 Introduction

Dermatophytosis is an infectious disease caused by fungi infecting keratinized tissue. Depending on their host adaption, they are divided into three groups, namely the human adapted anthropophilic dermatophytes, the animal adapted zoophilic dermatophytes, and the saprophytic geophilic dermatophytes [1]. However, species barriers can be crossed and under certain conditions, geophilic dermatophytes can also cause disease in humans and animals [1]. In pet rabbits, dermatophytosis is not very frequent, but due to its zoonotic potential, it still is an important infectious disease [2]. Young animals have a higher risk of infection, especially in combination with poor husbandry [3]. Typical lesions include alopecia, erythema, and pruritus, mostly in the face, on ears or legs [3]. Rabbits can also be asymptomatic carriers of dermatophytes [4,5]. The most common dermatophyte in rabbits is *Trichophyton mentagrophytes*, followed by *Microsporum canis* [5,6]. In addition, there have been reports about other dermatophytes isolated from rabbits, such as *Nannizzia fulva* (formerly *Arthroderma fulvum*) and *Trichophyton benhamiae* (formerly *Arthroderma benhamiae*) [7,8]. The identification of dermatophytes can be challenging, and one should not rely on a single aspect, but rather on a combination of different criteria such as host-spectrum, virulence, morphology, microscopy, metabolite production, mating behaviour, and molecular information [9]. Moreover, for molecular identification, more than one gene should be sequenced for a more accurate diagnosis [9]. However, the identification of dermatophytes is also complicated by the frequent changes in their nomenclature and the description of new species. Especially for geophilic and zoophilic dermatophytes, there are still numerous species that are yet to be taxonomically classified [9]. *Arthroderma* (*A.) lilyanum* is one of the recently described species and was isolated from two cats with clinical signs of dermatophytosis in the USA [10]. Hereby, the first isolation of *A. lilyanum* from a rabbit is reported. Following, the clinical signs, laboratory analysis and treatment are described.

## 2 Case presentation

**Fig. 1:**
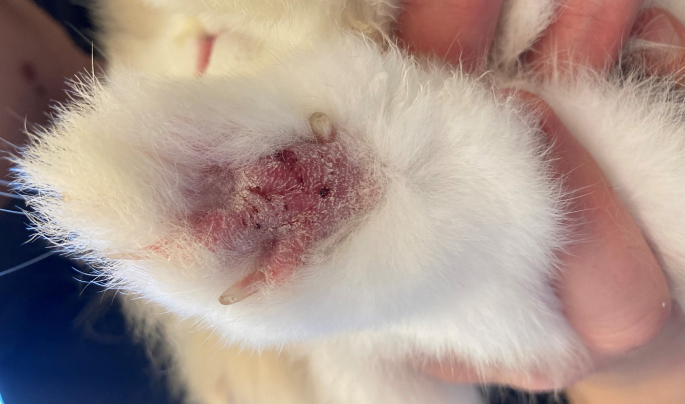
Routine check of a rabbit on day 0. A focal alopecic area associated with erythema, scaling, and few dark brown crusts in the palmar area of the right forelimb was revealed. (For interpretation of the references to colour in this figure legend, the reader is referred to the Web version of this article.)

**Fig. 2:**
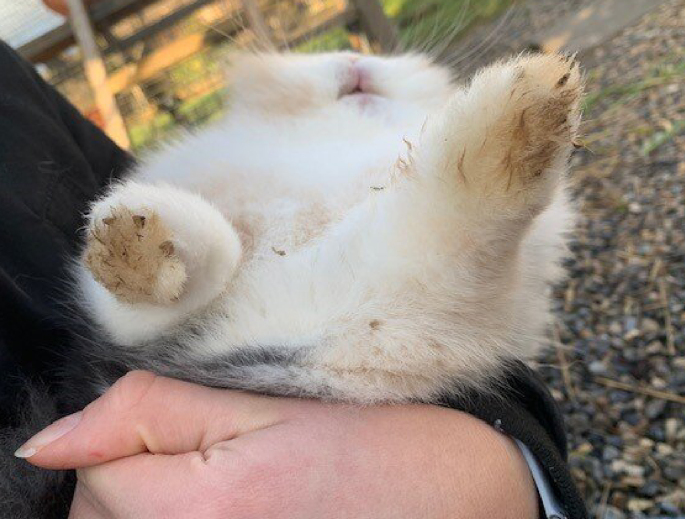
Healed paw one month after completing the treatment with octenidine dihydrochloride (day 44).

A two-year old male castrated lionhead rabbit, 2.17 kg, was presented for a routine check (day 0) before being rehomed to a new owner from an animal shelter. The animal keepers reported that the rabbit was healthy and in good general condition and lived in an outdoor cage on a natural hard ground with grit, sand, and some flagstones. Dermatologic examination revealed a focal alopecic area associated with erythema, scaling, and few dark brown crusts in the palmar area of the right forelimb (Fig. 1). Based on the history and clinical findings, a skin infection (bacterial or fungal), trauma due to extensive digging and early-stage ulcerative pododermatitis were considered the most likely differential diagnoses. Hair and skin scraping material were sampled for a mycological examination. Meanwhile the skin lesions were treated with octenidine dihydrochloride (Octenisept™, Schülke & Mayr AG, Zurich, Switzerland) twice daily for two weeks (until day 14). No clinical reassessment was performed since the rabbit was rehomed; however, the new owner shared a picture of the right forelimb in complete remission (Fig. 2) one month after completing the treatment (day 44).

**Fig. 3:**
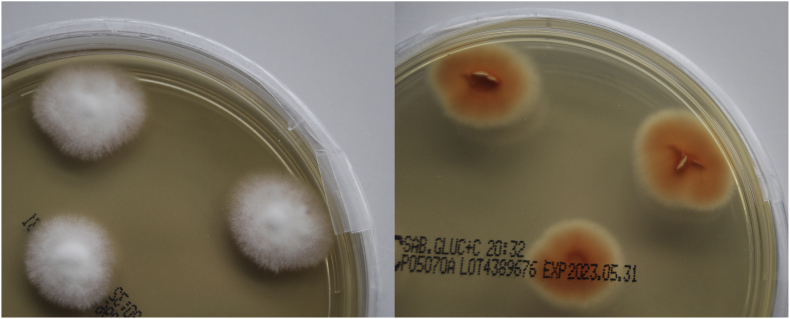
*Arthroderma (A.) lilyanum* one week after being subcultured on Sabouraud Glucose Chloramphenicol Selective Agar. Left: white surface colour. Right: orange reverse colour. (For interpretation of the references to colour in this figure legend, the reader is referred to the Web version of this article.)

**Fig. 4:**
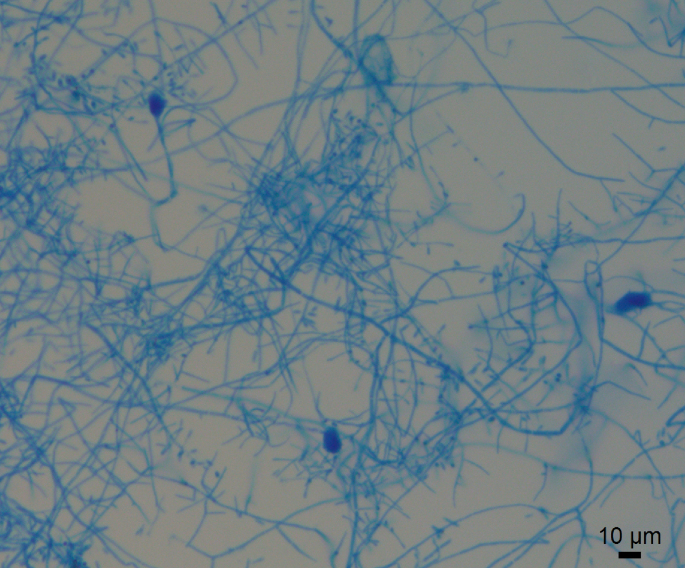
Microscopy of *A. lilyanum* one week after being subcultured, stained with lactophenol cotton blue stain. Septate hyphae and ellipsoidal microconidia are visible. 200X magnification. (For interpretation of the references to colour in this figure legend, the reader is referred to the Web version of this article.)

The clinical sample was inoculated on a Dermatophytes Selective Agar (Taplin, Thermo Scientific™, Waltham, MA, USA) for four weeks (until day 30) at room temperature (20 °C ± 2 °C). The plate was checked for fungal growth every week and after two weeks (day 14), fungal growth suspicious for dermatophytes was observed. Microscopy using lactophenol cotton blue stain was inconclusive; therefore, the fungus was subcultured on Taplin agar. After a week of incubation at room temperature (day 21), a white fungus was growing, and microscopy of the subculture was performed. Since the microscopy was still inconclusive, the fungus was subcultured on Sabouraud Glucose Chloramphenicol Selective Agar (Thermo Scientific™, Waltham, MA, USA). After another week of incubation at room temperature (day 28), a white colony was grown, with a rather orange colony reverse (Fig. 3). Microscopically, septate hyphae and ellipsoidal microconidia were visible (Fig. 4). Sequencing of the ITS and β-tubulin genes was carried out using previously published primers [11,12]. For DNA extraction, AllPrep® Fungal DNA/RNA/Protein Kit (Qiagen, Hilden, Germany) was used according to the manufacturer's manual. The PCR products were purified using the QIAquick® PCR Purification Kit (Qiagen, Hilden, Germany). Subsequently, the purified DNA was sent to Microsynth (Balgach, Switzerland) for Sanger sequencing. The resulting sequences were 633 bp long for ITS and 433 bp for β-tubulin, respectively. Sequence similarity search using BLAST (https://blast.ncbi.nlm.nih.gov/Blast.cgi accessed on January 11, 2023), showed the highest identity (100% for ITS and 99% for β-tubulin) with the sequences of isolate CSUCA018 (accession numbers OK597194 and OL342745, respectively), described by Moskaluk and VandeWoude as *A. lilyanum* [10].

## 3 Discussion

Here we report the clinical presentation, isolation, and identification of *A. lilyanum* in a pet rabbit. The novel species *A. lilyanum* was discovered in two 6-week-old cats in the USA [10]. Both cats were presented in 2019 with typical clinical signs of dermatophytosis, including scales, crusts, and alopecia on the abdomen and the head, respectively. Furthermore, one of the cats was coinfected with *Microsporum canis*. The detection of *A. lilyanum* in a rabbit shows that infection is not limited to cats, and it is likely to have a broader host range. However, it is not yet known whether this dermatophyte is also zoonotic. The lesion presented in this case report did show typical signs of dermatophytosis, such as alopecia, erythema, and crusts. However, while spread of dermatophyte lesions to paws can occur [13], the localisation at the palmar side of the forepaw in this case report was a rather uncommon localisation for a single and primary lesion of dermatophytosis. Hence, it is questionable, whether the lesion was caused by *A. lilyanum*. Rabbits can also be asymptomatic carriers of dermatophytes [4,5]. So far, the dermatophyte was only reported once, and further studies are needed to determine its pathogenicity and host range. Furthermore, younger animals are at a higher risk of infection from dermatophytes [3], but the rabbit in this case report was almost three years old, which could also explain the lack of more typical clinical signs.

A differential diagnosis for the lesion is ulcerative pododermatitis, which is the most frequent skin disease in pet rabbits [2]. This disease is caused by a pressure sore, typically occurring on the caudal aspect of the tarsus and metatarsus, though it can also affect the metacarpus [13]. The pressure sore can be caused by unsuitable flooring, obesity, or inactivity [13]. Furthermore, secondary bacterial infections can occur. Unfortunately, no bacterial culture was conducted in this case, thus, it is not known, whether a bacterial infection was also present. There is a description of mycotic pododermatitis in turkeys, which were infected by different environmental fungi [14]. Thus, it is possible, that an opportunistic infection of *A. lilyanum* occurred in this case. As it is not known whether *A. lilyanum* is geophilic or zoophilic [10], the occurrence of this dermatophyte in the environment is possible.

Dermatophytosis can be self-limiting, but treatment is recommended due to its zoonotic potential [3]. Topical antifungal agents are recommended for treatment of small and superficial lesions [3]. In addition, the affected animal should be separated from healthy animals and the environment should be cleaned thoroughly [3]. The treatment of ulcerative pododermatitis aims at relieving pressure, e.g. by more compliant flooring, analgesia, and treatment of secondary infections [13], which was not necessary herein as the lesions were only mild. In the case described here, the lesion was treated locally with octenisept. Octenisept is a disinfectant containing octenidine amongst others. According to the manufacturer, it is bactericidal and also effective against *Candida albicans* (https://www.schuelke.com/de-de/produkte/octenisept-Wund-Desinfektion.php accessed on 27.02.2023). Hence, octenisept might also be effective against other fungi, which might explain the rapid cure of the lesion in this case. Furthermore, if any secondary bacterial infection was present, octenisept might also have helped to cure it. The rabbit presented in this case report was moved from an animal shelter to a private household after presentation to the veterinarian, thus, no treatment of the environment was conducted. This change of environment might also have helped to cure the lesion, if ulcerative pododermatitis was part of its cause.

Since the first description of *A. lilyanum* was in the USA, and the second report in Switzerland, it is likely that this dermatophyte is much more widespread than previously known. One possibility for not diagnosing this dermatophyte before, could be a coinfection with other dermatophytes, that can overgrow *A. lilyanum* [10]. Furthermore, distinction of dermatophytes based on morphology and microscopy alone is difficult and sequencing of one single gene can also be insufficient for species identification [9]. Hence, animals infected with *A. lilyanum* might have been misdiagnosed with other dermatophytes in the past.

This is the first report of *A. lilyanum* in a rabbit and in Switzerland. At a routine check, an alopecic area on the forepaw of a rabbit was discovered. Since it is not clear whether the dermatophyte caused this clinical sign, it is possible that an infection with *A. lilyanum* in rabbits remains asymptomatic. Furthermore, this case report indicates that *A. lilyanum* is more widespread than previously thought and might infect various animal species. To safely identify the dermatophyte, sequencing the ITS and β-tubulin genes has shown to be a reliable option.

## Declaration of competing interest

There are none.
